# Evaluation after implementation of chemical bowel preparation for surgical site infections in elective colorectal cancer surgery and role of antimicrobial stewardship pharmacist: Retrospective cohort study

**DOI:** 10.1186/s40780-024-00333-1

**Published:** 2024-02-19

**Authors:** Yasuhiro Sasaki, Akira Kurishima, Chieko Miyamoto, Kenichiro Hataji, Toru Tezuka, Hideo Katsuragawa

**Affiliations:** 1 Department of Pharmacy, Tama-Nambu Chiiki Hospital, Tokyo Metropolitan Organization, 1-2, Nakazawa 2-Chome, Tama, Tokyo 206-0036 Japan; 2 Division of Public Health, Teikyo University Graduate School of Public Health, 2-11, Kaga 1-Chome, Itabashi-ku, Tokyo 173-8605 Japan; 3 Department of Nursing, Tama-Nambu Chiiki Hospital, Tokyo Metropolitan Organization, 1-2, Nakazawa 2-Chome, Tama, Tokyo 206-0036 Japan; 4 Department of Surgery, Tama-Nambu Chiiki Hospital, Tokyo Metropolitan Organization, 1-2, Nakazawa 2-Chome, Tama, Tokyo 206-0036 Japan; 5 Department of Endoscopy, Tama-Nambu Chiiki Hospital, Tokyo Metropolitan Organization, 1-2, Nakazawa 2-Chome, Tama, Tokyo 206-0036 Japan

**Keywords:** CHEMICAL bowel preparation, Surgical site infection, Antimicrobial stewardship pharmacist

## Abstract

**Background:**

We evaluated the predictive factors for surgical site infections (SSIs) in elective colorectal cancer surgery and the role of antimicrobial stewardship (AS) pharmacists in modifying the clinical pathway.

**Main body:**

Between February 2017 and January 2022, 414 elective colorectal cancer surgeries were performed. The results of multivariate analysis by SSI incidence were adjusted odds ratio (aOR): 0.45; 95% confidence interval (CI): 0.22–0.96 (*P* = 0.039) for sex (female), aOR: 0.27; 95% CI: 0.13–0.58 (*P* < 0.001) for laparoscopy, aOR: 0.42; 95% CI: 0.19–0.91 (*P* = 0.029) for chemical bowel preparation. The median (interquartile range) postoperative length of stay was 12 (10.0–18.5) vs. 10 (9.0–13.0) days before and after the clinical pathway was modified (*P* < 0.001).

**Conclusion:**

The role of AS pharmacists was primarily to conduct a literature search to explore whether SSIs could be ameliorated by pharmacotherapy, coordinate the addition of chemical bowel preparation, and epidemiologically confirm their effectiveness.

**Supplementary Information:**

The online version contains supplementary material available at 10.1186/s40780-024-00333-1.

## Background

In 2016, the Japanese Society of Chemotherapy and the Japan Society for Surgical Infection jointly published practical guidelines for the use of prophylactic antibiotics to prevent postoperative infections [1]. Although parallel efforts by pharmacists toward appropriate prophylactic antibiotic use have been reported [2], reports on pharmacists’ efforts to prevent surgical site infections (SSIs) are scarce [3].

SSI prevention strategies include blood glucose control, normothermia maintenance [4], appropriate antimicrobial agent selection, timing of administration, and duration of therapy. It is recommended that prophylactic antimicrobial agents be combined with oral antimicrobial agents [1]. However, according to the results of a survey conducted in 2015, few facilities in Japan have adopted this approach [5].

At Tokyo Metropolitan Organization Tama-Nambu Chiiki Hospital, a pharmacist began providing full-time antimicrobial stewardship (AS) services in 2019. Prior to this, the pharmacist sorted out the in-hospital adoption of intravenous antimicrobial agents, post-prescription audits and feedback for broad-spectrum antimicrobial agents [6], as well as actively intervene in blood culture-positive patients [7]. Mid- to long-term intervention with intravenous antimicrobial agents decreases the resistance rate of *Pseudomonas aeruginosa* [8]. AS pharmacists reconsidered their SSI countermeasures after receiving a report from an infection control nurse (ICN), stating that SSI incidence in patients undergoing colorectal cancer surgery was high.

This study aimed to evaluate the efficacy of additional chemical bowel preparation for SSI prophylaxis in elective colorectal cancer surgery and report the role of dedicated AS pharmacists in modifying the clinical pathway.

## Main text

This retrospective cohort study was conducted at Tokyo Metropolitan Organization Tama-Nambu Chiiki Hospital, a 287-bed secondary emergency medical care facility in Tama, Tokyo, Japan, with no infectious disease physicians. Patients who underwent elective colorectal cancer surgery between January 2017 and January 2022 were included. Exclusion criteria included cases involving multiple surgeries. Modifications to the clinical pathway were implemented in June 2019. SSIs were evaluated in 414 cases (224 before and 190 after clinical pathway modification) based on sex, age, diabetes status, smoking status, surgical site (colon or rectum), wound class, American Society of Anesthesiologists physical status (ASA-PS) classification [9], laparoscopic surgery, stoma construction, mechanical bowel preparation, and chemical bowel preparation. SSIs were assessed from medical records by the ICN based on these criteria (Supplement 1) [4].

Potential risk factors associated with SSIs for each procedure type were assessed using univariate modeling analysis. Categorical variables were compared using the x^2^ test. Variables with a *p*-value < 0.2 in the univariate modeling analysis were considered potential independent variables and were entered into the logistic regression model. A multivariate model was developed using a forward stepwise logistic regression. Variables were retained in the final model if the 2-tailed *p*-value < 0.05. The Mann–Whitney *U* test was performed for postoperative hospital stay and total medical fees. Statistical analysis was performed using JMP, version 14.2.0 (JMP). The Institutional Review Board of Tokyo Metropolitan Tama-Nambu Chiiki Hospital approved this study.

Table 1 shows the patient characteristics. SSI incidence during the study period was 9.9% (41 of 414 cases). Univariate logistic regression analysis demonstrated statistical significance for sex (*P* = 0.018), smoking status (*P* = 0.025), wound class (*P* = 0.007), ASA-PS (*P* = 0.114), laparoscopy (*P* < 0.001), and chemical bowel preparation (*P* = 0.012). The results of multivariate logistic regression analysis adjusting for sex, smoking status, wound class, ASA-PS, laparoscopy, and chemical bowel preparation showed sex (female) (adjusted odds ratio [aOR], 0.45 [95% CI, 0.22–0.96], *P* = 0.039), laparoscopy (aOR, 0.27 [95% CI, 0.13–0.58], *P* < 0.001), and chemical bowel preparation (aOR 0.42 [95% CI, 0.19–0.91], *P* = 0.029) (Table 2). The SSI incidence rates were 12.5% (28 of 224 cases) before modification and 6.8% (13 of 190 cases) after. The median (interquartile range [IQR]) postoperative hospital stay was 12 days (10.0–18.5) before modification and 10 days (9.0–13.0) after (statistically significant; *P* < 0.001). The total medical fee (IQR) was 1,490,620 yen (1,291,080–1,779,790) before modification and 1,390,110 yen (1,291,460–1,676,230) after (*P* = 0.066). Adverse reactions after mechanical and chemical bowel preparations included anaphylactic shock (1 case).

**Table 1:** Baseline and clinical characteristics of the study population analyzed using Pearson's chi-square test

Characteristics, n (%)	Patient undergoing elective colorectal surgery (*N* = 414)		*p*
	SSI ( +) (*N* = 41)	SSI (-) (*N* = 373)	
Female sex	13 (31.7%)	191 (51.2%)	0.018
Age ≥ 60	35 (85.4%)	322 (77.8%)	0.865
Diabetes	10 (24.4%)	64 (17.2%)	0.251
Smoking	11 (26.8%)	51 (13.7%)	0.025
Location			
Colon	31 (75.6%)	269 (72.1%)	0.635
Rectum	10 (24.4%)	104 (27.9%)	
Wound class			
Clean-contaminated	39 (95.1%)	371 (99.5%)	0.007
Dirty-infected	2 (4.9%)	2 (0.5%)	
ASA-PS			
1	1 (2.4%)	38 (10.2%)	0.114
2	35 (85.4%)	312 (83.7%)	
3	5 (12.2%)	23 (6.2%)	
Laparoscopy	26 (63.4%)	316 (84.7%)	< 0.001
Stoma construction	8 (19.5%)	54 (14.5%)	0.391
Mechanical bowel preparation	30 (73.2%)	267 (71.6%)	0.830
Chemical bowel preparation	9 (2.2%)	158 (38.2%)	0.012

*SSI* surgical site infection, *ASA-PS* American Society of Anesthesiologists physical status

**Table 2:** Multivariate analysis for factors independently associated with surgical site infection in colorectal surgery

Characteristic	Adjusted odds ratio (95% CI)
Female sex	0.45 (0.22–0.96)
Laparoscopy	0.27 (0.13–0.58)
Chemical bowel preparation	0.42 (0.19–0.91)

Bivariate analyses were analyzed using Fisher's exact tests. Variables with a *p* < 0.20 by bivariate analysis were included in multivariable model selection. Model selection was conducted using stepwise logistic regression and consideration of 2-way interaction terms. The level of significance was set at α = 0.05

*Abbreviation*: *CI* confidence interval

**Fig. 1:**
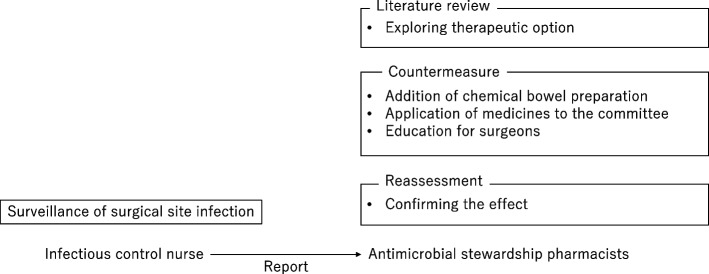
Role of antimicrobial stewardship pharmacists in preventing surgical site infections in elective colorectal surgery

The role of AS pharmacists was to explore whether SSIs in patients undergoing elective colorectal cancer surgery could be ameliorated with pharmacotherapy, to coordinate with the relevant departments, and to educate surgeons (Fig. 1). Adding chemical bowel preparation to prophylactic antimicrobials in elective colorectal cancer surgery has been reported to decrease SSI incidences [10]. In response, AS pharmacists decided to add chemical bowel preparations (a single dose of kanamycin [1000 mg] and metronidazole [750 mg] administered orally at 18:00 and 22:00 the day before surgery) and shorten the duration of intravenous antimicrobial use from 48–72 h post-surgery to ≤ 1 day post-surgery. The usefulness of the chemical bowel preparation, expected side effects, and modifications in the clinical pathway were explained by the AS pharmacist and approved at the surgical conference. When adding chemical bowel preparation, an application for adopting a new agent for kanamycin was submitted to the Pharmaceutical Affairs Committee and an off-label application to the Ethics Review Committee by an AS pharmacist in advance. Finally, chemical bowel preparation was added in June 2019.

AS pharmacists have led the addition of chemical bowel preparation and shortened intravenous antibiotic administration in the clinical pathway for elective colorectal cancer surgery. Chemical bowel preparation, sex, and laparoscopy were the relevant exploratory factors for SSIs. After modifications, the number of postoperative hospital stays was reduced and SSI incidence was lower. Since shortening the intravenous antibiotic administration time has no effect on SSIs [11], this is unlikely to contribute a lower SSI incidence. The role of AS pharmacists in adding chemical bowel preparation ranged from the exploration of effective pharmacotherapy for SSI prevention to the epidemiological verification of its effectiveness. AS pharmacists taking the initiative to change clinical pathways may contribute to the efficacy of medical therapies.

As pharmacotherapy is the core of treatment, pharmacists should take the initiative to change or evaluate treatment [12]. The clinical pathways through which pharmacists take the initiative have been reported for perioperative thromboprophylaxis [13] and postoperative pain relief [14]. In Japan, reports exist on the clinical pathways taken by pharmacists, including the development of criteria for treatment after surgery to standardize medical care [15] and supportive care for cancer chemotherapy [16]; however, similar efforts for SSI prevention do not exist. The role of pharmacists in clinical pathways for SSIs may not be recognized in Japan, given that the main methods to prevent SSIs are normothermia, appropriate antiseptic use, and postoperative blood glucose control [17]. The AS pharmacist introduced chemical bowel preparation after a literature review was triggered by ICN surveillance reports. AS pharmacists should regularly communicate with other professionals and to determine whether they can contribute to pharmacotherapy. Deciding in advance the appropriate antimicrobial selection and administration timing by pharmacists and educating physicians is the simplest way to resolve the inappropriate selection and timing of perioperative antimicrobials [18, 19] that many institutions experience.

Risk factors for SSIs in elective colorectal cancer surgery include chemical bowel preparation [20], use of laparoscopy [21], and sex [22]. In this study, adjustable chemical bowel procedures decreased the incidence of SSIs. The confirmation of the expected effects is another important task for pharmacists. The use of laparoscopy to reduce SSI incidence has also been reported [23]. Therefore, the appropriateness of laparoscopy should be evaluated for each institution. Similar to the rates in our study, 77.5% of laparoscopic procedures are performed in the rectum and 61.5% in the colon [24]. The association regarding sex is well known, but the reasons are not known [22].

This study has several limitations. First, this was a retrospective cohort study, and confounding factors that have not been investigated may have reduced the efficacy of chemical bowel preparation for SSIs. Certain risk factors associated with SSI, such as perioperative hyperglycemia, hypothermia, and operative time, were not examined in this study. Second, SSIs were assessed from medical records by the ICN, and may be underestimated. However, previous studies have reported similar results [25]. Third, whether the role of AS pharmacists is transferable to other facilities is uncertain because of differences between stakeholders.

## Conclusions

AS pharmacists led addition of chemical bowel preparation in the clinical pathway for elective colorectal cancer surgery. Our results showed that sex, laparoscopy use, and chemical bowel preparation were prognostic factors of SSI. This study is the first report of AS pharmacists in Japan improving the SSI rate by modifying the clinical pathway. The role of AS pharmacists was primarily to conduct a literature search to explore whether SSIs could be ameliorated by pharmacotherapy, coordinate the addition of chemical bowel preparation, and epidemiologically confirm their effectiveness.

### Supplementary Information

**Additional file 1.**

## Data Availability

The datasets used and/or analyzed in the current study are available from the corresponding author upon reasonable request. Transparency declarations: All the authors report no potential conflicts of interest.

## Abbreviations

AS: Antimicrobial stewardship
SSI: Surgical site infection
aOR: Adjusted odds ratio
IQR: Interquartile range
CI: Confidence interval
ICN: Infection control nurse
ASA-PS: American Society of Anesthesiologists Physical Status
