# Supplementary material for: Evaluation after implementation of chemical bowel preparation for surgical site infections in elective colorectal cancer surgery and role of antimicrobial stewardship pharmacist: Retrospective cohort study
Source: J Pharm Health Care Sci. 2024 Feb 19;10:11. doi: 10.1186/s40780-024-00333-1 (PMC10875839; doi:10.1186/s40780-024-00333-1)
Supplement: Supplementary file 1 — Additional file 1. [file 40780_2024_333_MOESM1_ESM.docx]

Supplement 1. Surgical Site Infection Criteria

Superficial incisional Surgical Site Infection (SSI) must meet the following criteria:

Date of event occurs within 30 days following the operative procedure

AND

involves only skin and subcutaneous tissue of the incision

AND

patient has at least one of the following:

A. purulent drainage from the superficial incision.

B. organism(s) identified from an aseptically-obtained specimen from the superficial incision or subcutaneous tissue by a culture or nonculture based microbiologic testing method which is performed for purposes of clinical diagnosis or treatment.

C. a superficial incision that is deliberately opened by a surgeon, physician or physician designee and culture or non-culture based testing of the superficial incision or subcutaneous tissue is not performed

AND

patient has at least one of the following signs or symptoms: localized pain or tenderness; localized swelling; erythema; or heat.

D. diagnosis of a superficial incisional SSI by a physician or physician designee

Deep incisional SSI must meet the following criteria:

Date of event occurs within 1 year postoperatively for surgeries in which an implant was inserted, or within 30 days postoperatively for surgeries in which an implant was not inserted.

AND

involves deep soft tissues of the incision (for example, fascial and muscle layers)

AND

patient has at least one of the following:

A. purulent drainage from the deep incision.

B. a deep incision that is deliberately opened or aspirated by a surgeon, physician or physician designee or spontaneously dehisces

AND

organism(s) identified from the deep soft tissues of the incision by a culture or non-culture based microbiologic testing method which is performed for purposes of clinical diagnosis or treatment or culture or nonculture based microbiologic testing method is not performed. A culture or non-culture based test from the deep soft tissues of the incision that has a negative finding does not meet this criterion.

AND

patient has at least one of the following signs or symptoms: fever (>38°C); localized pain or tenderness.

C. an abscess or other evidence of infection involving the deep incision detected on gross anatomical exam, histopathologic exam, or imaging test.

Organ/Space SSI must meet the following criteria:

Date of event occurs within 1 year postoperatively for surgeries in which an implant was inserted, or within 30 days postoperatively for surgeries in which an implant was not inserted.

AND

involves any part of the body deeper than the fascial/muscle layers that is opened or manipulated during the operative procedure

AND

patient has at least one of the following:

A. purulent drainage from a drain placed into the organ/space (for example, closed suction drainage system, open drain, T-tube drain, CT guided drainage).

B. organism(s) identified from fluid or tissue in the organ/space by a culture or non-culture based microbiologic testing method which is performed for purposes of clinical diagnosis or treatment.

C. an abscess or other evidence of infection involving the organ/space detected on gross anatomical exam or histopathologic exam, or imaging test evidence definitive or equivocal for infection.
